# Gut microbiota dysbiosis remodels the multi-tissue transcriptional landscape of the host

**DOI:** 10.1186/s40168-026-02495-6

**Published:** 2026-08-14

**Authors:** Liang Huang, Shuqi Liu, Sai Xiao, Yige Li, Shiqi Luo, Erjia Hou, Yizhen Wang, Xin Zong

**Affiliations:** 1 Key Laboratory of Molecular Animal Nutrition, Ministry of Education, College of Animal Sciences, Zhejiang University, Hangzhou, 310058 PR China; 2 Key Laboratory of Animal Nutrition and Feed Science in Eastern China, Ministry of Agriculture, College of Animal Sciences, Zhejiang University, Hangzhou, 310058 PR China; 3 Zhejiang Key Laboratory of Nutrition and Breeding for High-Quality Animal Products, College of Animal Sciences, Zhejiang University, Hangzhou, 310058 PR China

**Keywords:** Cross-tissue, Multi-omics, Machine learning, Gut-liver axis

## Abstract

**Background:**

The gut microbiota exerts a profound influence on host physiology, but its systemic impact on gene expression across diverse tissues remains poorly characterized.

**Results:**

This study investigated the transcriptional effects of gut microbiota depletion and restoration in mice across six tissues (colon, jejunum, liver, heart, lung, and kidney) using whole-transcriptome sequencing. We found that the presence of gut microbiota significantly altered the transcriptome, with the most pronounced effects in the colon. Using a linear mixed-effects model, we identified 7365 candidate microbiota-responsive genes. Tissue-specific analysis revealed that these genes were associated with distinct functional pathways, such as immunity in the gut and lung, and metabolism in the liver. Further refinement with LASSO regression pinpointed candidate microbiota‑responsive genes, whose ortholog expression levels were significantly associated with patient survival in corresponding human cancers (e.g., LIHC, LUAD, KIRC). Furthermore, we observed a widespread remodeling of competing endogenous RNA (ceRNA) networks by the gut microbiota. Single-cell data analysis highlighted a potential gut-liver axis interaction, primarily mediated by colonic enterocytes and hepatic cholangiocytes, meanwhile gut microbiota repressed the transcription initiation of *Noct* in colonic enterocytes, whose expression level was significantly negatively correlated to gut-liver axis interaction.

**Conclusions:**

Our findings provide a comprehensive map of the multi-tissue transcriptional landscape shaped by the gut microbiota, which regulate immune response of the gut-liver axis according to inhibit the transcription of *Noct*, revealing tissue-specific regulatory mechanisms and identifying key genes with potential clinical relevance in cancer.﻿

Video Abstract

**Supplementary Information:**

The online version contains supplementary material available at https://doi.org/10.1186/s40168-026-02495-6.

## Introduction

The recognition of the gut microbiota as a metabolic “organ” stems from its mediation of essential processes like bile acid and fatty acid metabolism [1–5]. The metabolites produced through these pathways play a critical role in regulating host immunity, metabolism, digestion, and absorption [6–10]. The gut microbiota and intestinal tissue interact reciprocally, maintaining a dynamic equilibrium, disruption of this balance is a known etiology of intestinal disorders [11–13]. Furthermore, by using the gut as a conduit, the microbiota regulates inter-tissue signaling axes, thereby modulating immune homeostasis in peripheral tissues [14–16]. It is particularly noteworthy that the gut-liver axis serves as a pivotal hub connecting the digestive, immune, and metabolic systems [17–19]. It underscores the liver and the intestine—particularly its resident microbiota—as an integrated functional whole that collectively maintains organismal health [20–22]. Yet, a multi-tissue understanding of how the gut microbiota coordinates transcriptomic responses is lacking, particularly regarding the precise gene networks and mechanisms responsible for tissue-specificity.

Shen et al. employed multi-omics approaches, including single-cell and spatial transcriptomics, to investigate the effects of gut microbiota depletion on multiple organs [23], uncovering impacts on tissue morphology, immune cell composition, and metabolism such as bile acid and lipids. Their work highlighted the critical role of the ZBTB20-LPL axis in lipid regulation; however, a comprehensive analysis of global changes across tissues was lacking. Here, using whole-transcriptome sequencing, we systematically characterize how microbiota depletion and subsequent restoration reshape the transcriptomic landscapes across multiple host tissues, generating the transcriptomic atlas across tissues under different conditions. Capitalizing on the widespread application of machine learning in transcriptomics for clinical and predictive purposes [24–27], we employed it to screen for and identify tissue-specific candidate microbiota-responsive genes modulated by the gut microbiota. Furthermore, we mapped the ceRNA regulatory network reshaped by the gut microbiota. Moreover, by leveraging public single-cell RNA-seq and ATAC-seq datasets, we delineate the immune regulatory effects of the gut microbiota along the gut-liver axis.

Collectively, this study constructed a panoramic atlas of gut microbiota-regulated transcription across multiple tissues, revealing intricate, tissue-specific mechanisms. It serves as a rich gene resource and provides a novel framework—integrating multi-omics and machine learning—for understanding the systemic impact of host-microbiota interactions on physiology and diseases like cancer.

## Results

### Disturbed transcripts by gut microbiota in multi tissues

**Fig. 1:**
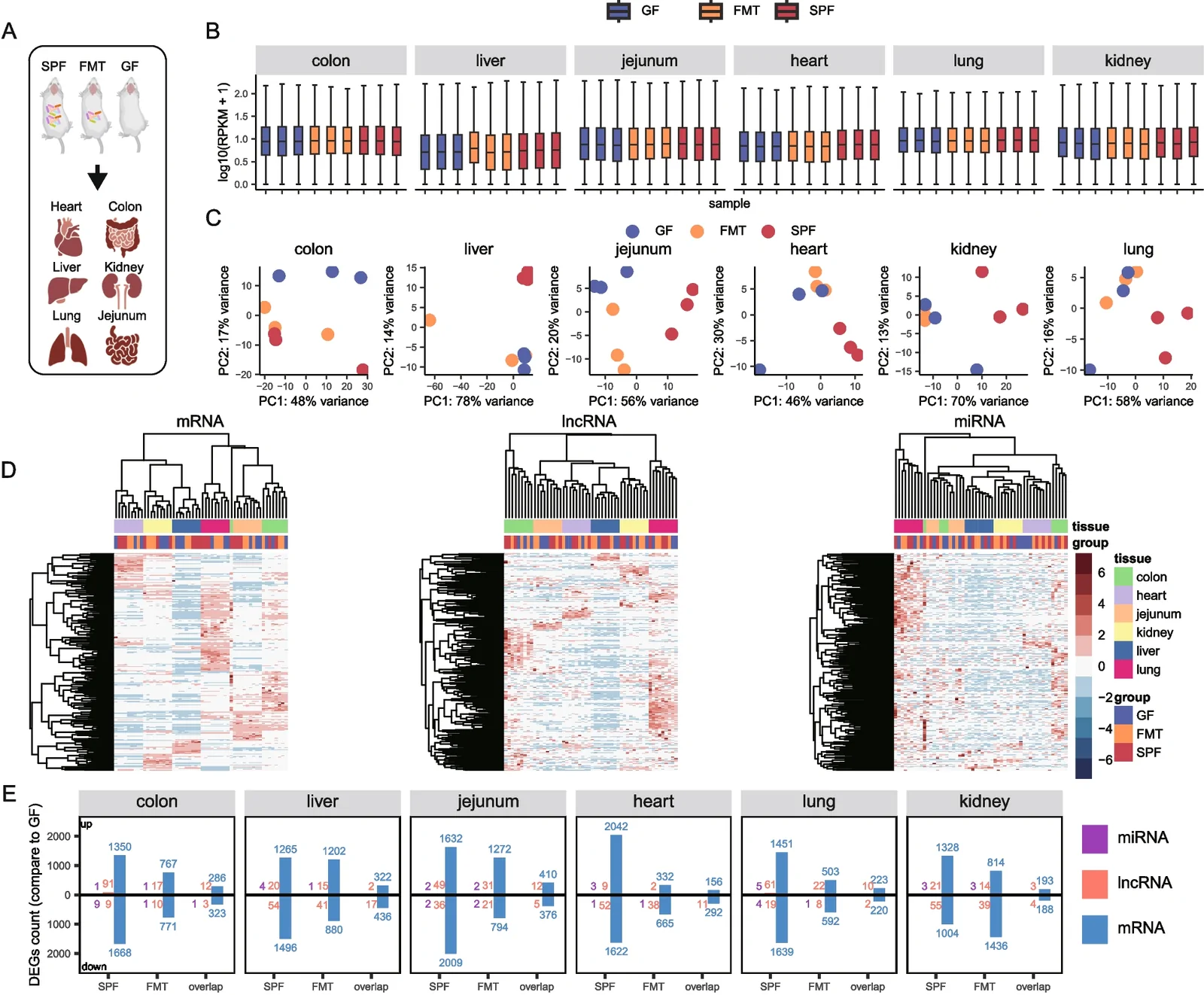
Gut microbes disturb multi-tissue transcripts. **A** Design of the research. **B** Distribution of per‑gene RPKM (log₁₀‑transformed) across all genes in each sample. **C** Principal component analysis (PCA) of transcriptome in each tissue. **D** Heatmap of expression of transcripts, including mRNA and non-coding RNA. **E** Counts of DEGs in each tissue. The columns above y = 0 indicate the number of upregulation, and the columns below y = 0 indicate the number of downregulation. Different colors represent RNA types

To explore the effect of gut microbiota on the process of transcription in multi tissue, we performed whole transcriptome sequencing using mice under three conditions (GF, FMT and SPF) as models (Fig. 1A), involve 6 tissues (colon, liver, jejunum, heart, lung and kidney), 9 samples per tissue (total 54 samples), and produced almost 718 Gb of data. Statistical analysis show that whole transcripts expression level is not affected by the gut microbiota in most of these tissues except colon (Fig. 1B). Under SPF condition, transcription is enhanced in the colon of mice. Corresponding to this, PCA showed significant distance between GF mice and non-GF (SPF and FMT) mice (Fig. 1C, Fig. S1), while a similar result also appeared to jejunum. While the gut microbiota was restored via FMT, its transcriptional influence appeared limited to the gut, as peripheral tissues (liver, heart, lung, kidney) of SPF mice remained distinct from those in other conditions. Further, to detect the impact of gut microbiota on tissues’ function, we screened differentially expressed genes (DEGs), including coding and non-coding genes, of tissues between GF mice and non-GF mice, and found the intersected DEGs between FMT and SPF (Fig. 1D, E). The up-regulated and down-regulated genes from these intersections were subjected to KEGG pathway enrichment analysis, respectively (Fig. S2). It turned out that immunity related pathways, like PI3K−Akt signaling pathway, Cytokine−cytokine receptor interaction, and NOD−like receptor signaling pathway, are up-regulated in gut, while carbon metabolism, fructose and mannose metabolism and pyruvate metabolism were down-regulated in gut, implying the presence of the gut microbiota enhances the host’s immune response and partially compensates for the host’s catabolism of carbohydrates. Moreover, the presence of gut microbiota enhanced immunity of peripheral tissues as immune related pathways had been strengthened, such as PI3K−Akt signaling pathway in heart, Cytokine−cytokine receptor interaction and Toll−like receptor signaling pathways in lung, Phagosome pathway in kidney.

### Tissue-specific candidate microbiota‑responsive genes atlas

**Fig. 2:**
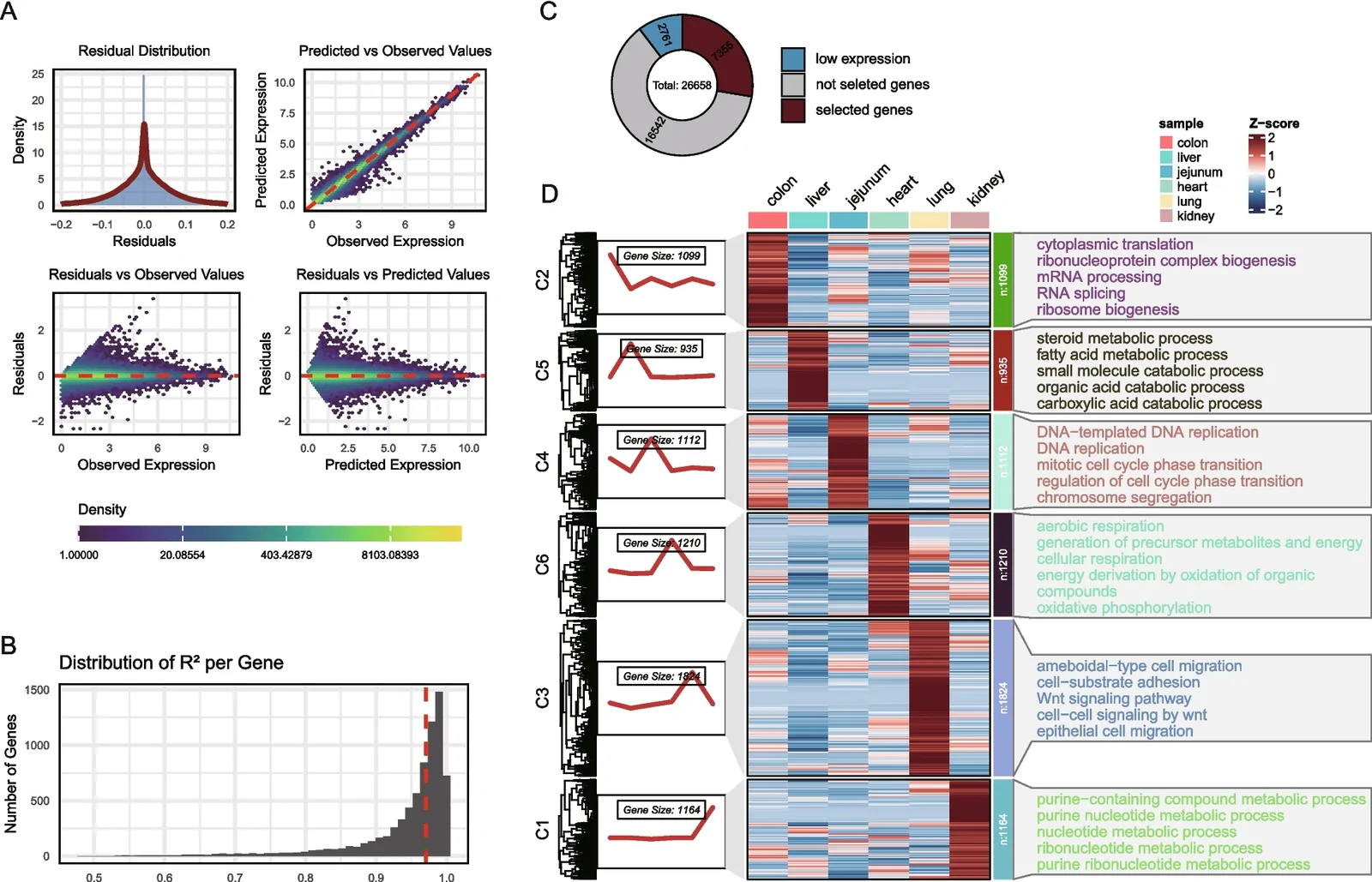
Tissue-specific candidate microbiota‑responsive genes atlas. **A** Comprehensive evaluation of Linear mixed effects model (LMM). MAE = 0.059893, RMSE = 0.102666, R^2^ = 0.994515. **B** Distribution of R^2^ per gene. Red line represents median value of R^2^, whose value above 0.96. **C** Result of LMM, which screens genes responded to gut microbes and tissues mix effect. **D** Complex heatmap of tissue specific genes expression and their enriched pathways in biological process (BP)

Since DEGs and their pathway enrichment results offered limited insight into the host’s tissue-specific responses to the gut microbiota, we turned to a machine learning strategy to precisely identify these genes. Utilizing machine learning, linear mixed-effects model (LMM), we examined genes that respond to gut microbiota in each tissue. With high confidence of the model (normal distribution of residuals, the slope of the ratio of predicted value to true value is about 1 and R^2^ exceed 0.96) (Fig. 2A, B), we finally screened 7365 genes from 26,658 genes (Fig. 2C). Given that the candidate genes from the LMM respond to tissue and gut microbiota, we clustered them with Mfuzz in order to identify tissue-specific response genes. As expected, genes in each cluster showed highly tissue-specific expression (Fig. 2D). Functional annotation (Biology Function), whose top 5 are shown, of the response genes for each tissue further revealed their functional specify respond to gut microbiota—the presence of gut microbiota affect the process of colonic transcription and translation, hepatic catalytic metabolism, jejunal cell cycle, cardiac oxidative phosphorylation, pulmonary cellular immunity and adhesion and renal purine metabolism. These function annotations suggest that the model-screened genes predominantly reflect tissue-specific rather than microbiota-related responses, the gene sets require further refinement to isolate the relevant signals.

### Tissue-specific candidate microbiota‑responsive genes and their behavior in prognosis

**Fig. 3:**
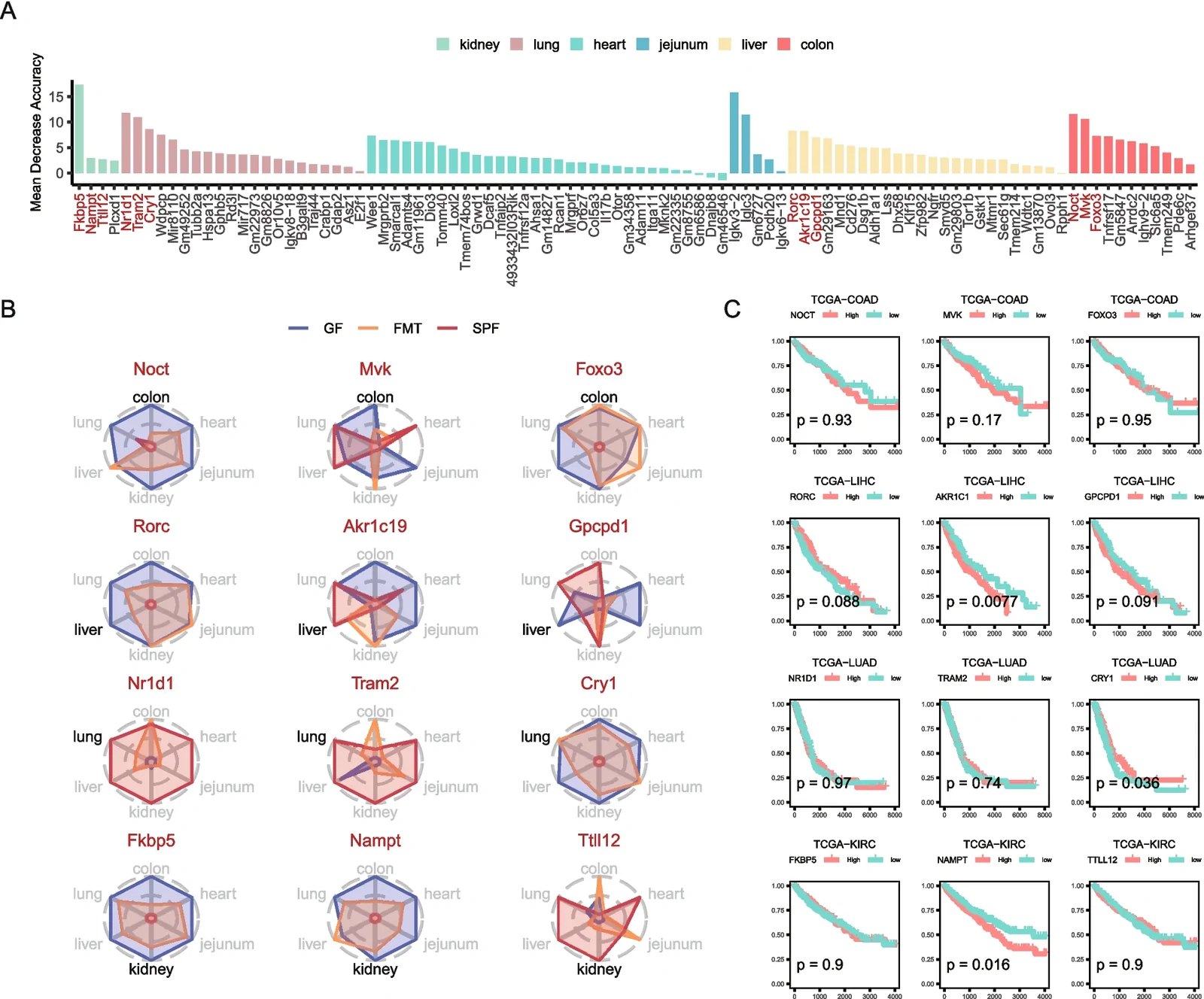
Tissue-specific candidate microbiota‑responsive genes and their behavior in tissue cancer. **A** Importance rank of candidate microbiota-responsive genes screened according LASSO model in each tissue, genes were ranked based on mean decrease accuracy according Randomforest model. Top 3 genes in colon, liver, lung and kidney are marked in red color. **B** Expression level of top 3 genes displayed in a way of radar chart. **C** Survival analysis of top 3 genes. Red represents high expression, while green represents low expression

The LASSO regression model is particularly suited for translational research, such as biomarker discovery and constructing polygenic predictive scores. Following the integration of the SPF and FMT groups into non-GF group, the genes identified by the LMM were subjected to LASSO regression. Using the contrast between GF and non-GF groups as the training condition, we successfully identified gut microbiota-responsive genes in each tissue (Fig. S3). Leveraging the importance scores derived from the random forest model, we ranked the screened genes based on their relative importance (Fig. 3A). The top 3 protein-coding genes from each tissue were selected for validation. Each exhibited responsiveness to the gut microbiota, as their expression under non-GF conditions was opposite to that in GF conditions, both in their native tissue and often in others (Fig. 3B). As we observed that the presence of gut microbiota had effect on the immunity of both peripheral tissues, we wonder how these genes behave on cancer. Based on database availability, we ultimately selected cancers corresponding to colon, liver, lung, and kidney tissues—represented by the COAD (Colon Adenocarcinoma), LIHC (Liver Hepatocellular Carcinoma), LUAD (Lung Adenocarcinoma), and KIRC (Kidney Renal Clear Cell Carcinoma) datasets, respectively—for survival analysis, as no matching cancer types were available for heart and jejunum tissues. The results showed that the expression of specific gut microbiota-responsive genes—*AKR1C1* (human ortholog of mouse *Akr1c19*, *P* = 0.0077) in liver, *CRY1* (*P* = 0.036) in lung, and *NAMPT* (*P* = 0.016) in kidney—had a significant impact on the survival rates of patients with the matching cancer types (Fig. 3C).

### Gut microbiota remodels tissue-specific ceRNA interaction network

**Fig. 4:**
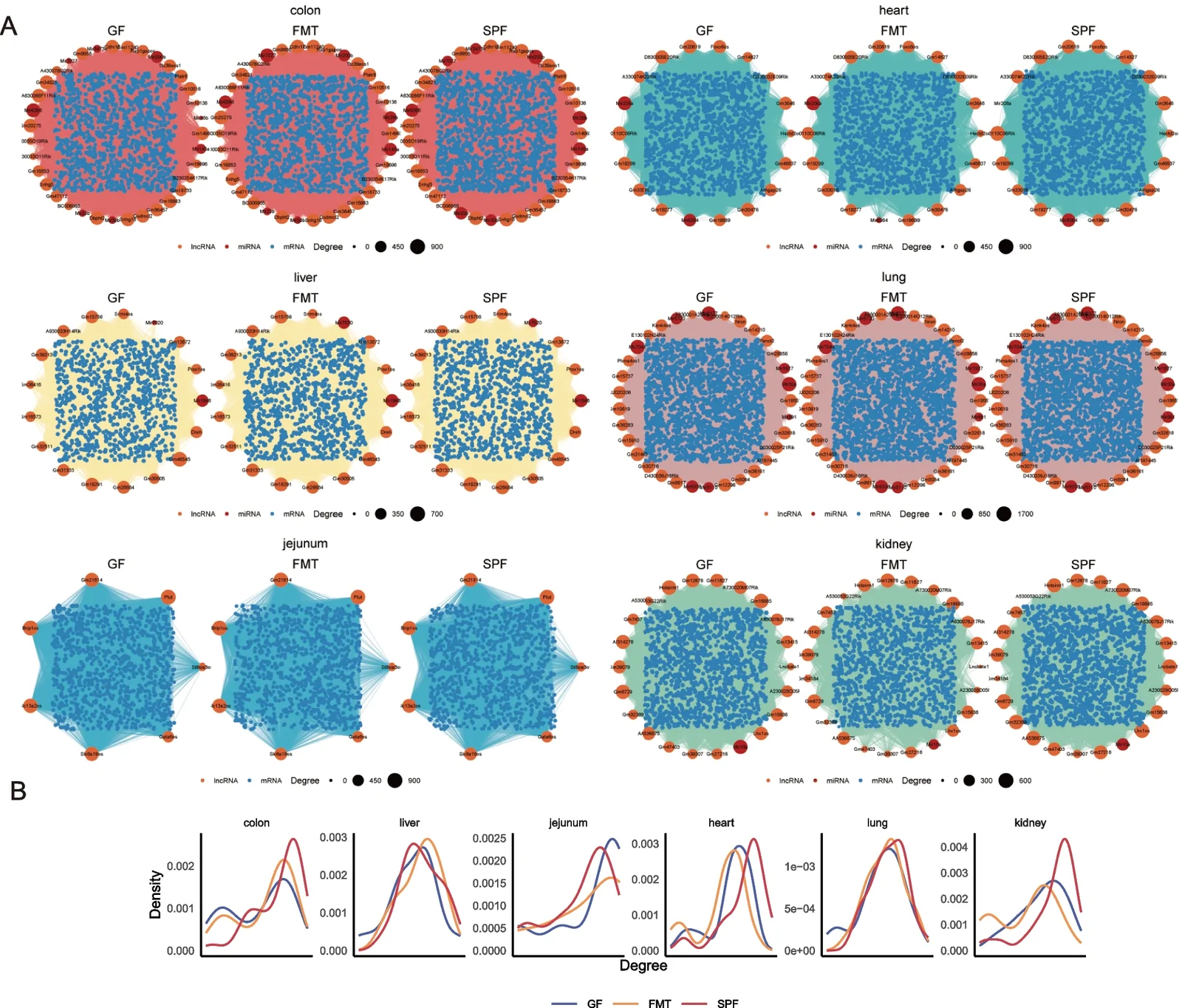
Tissue-specific ceRNA interaction network. **A** ceRNA network of each tissue. Non-coding mRNA are arranged in circular pattern, protein coding mRNA are arranged in square pattern. **B** Intensity of non-coding mRNA’s degree in each tissue

A key advantage of whole-transcriptome sequencing is its capacity to profile both coding and non-coding RNAs. These RNAs can form competing endogenous RNA (ceRNA) networks, which maintain the homeostasis of gene expression and thereby enable adaptation to environmental changes. Given this advantage, we therefore focused on investigating how the presence of gut microbiota regulates the ceRNA network within the host. Using LMM‑screened mixed‑effects genes, we constructed ceRNA networks in each tissue (Fig. 4A) via Spearman correlation. To visualize the differences in the ceRNA networks across various tissues and conditions, we constructed network maps by fixing the coordinates of each RNA and representing their connectivity by scaling the point size according to its degree. This visualization revealed distinct network architectures. For instance, in the colon of non-GF mice, we observed a significant increase in the number of regulated protein-coding genes. Non-coding RNA, especially miRNA, plays a role of messenger in ceRNA regulatory network. To further quantify the regulatory strength of non-coding RNAs within these networks under different conditions, we plotted density curves of their degrees (Fig. 4B). The results demonstrated that the presence of gut microbiota led to a marked increase in the connectivity of non-coding RNAs in the colon, jejunum, heart, and kidney, while it caused a decrease in the liver but a slight increase in the lung. Furthermore, we also detailed the ceRNA networks of candidate microbiota‑responsive genes across the various environmental conditions for each tissue (Fig. S4). These data point to a broad, microbiota-driven remodeling of ceRNA networks in multiple tissues, particularly within the intestinal system.

### scRNA-seq reveal the effect of gut microbes on liver-gut axis

**Fig. 5:**
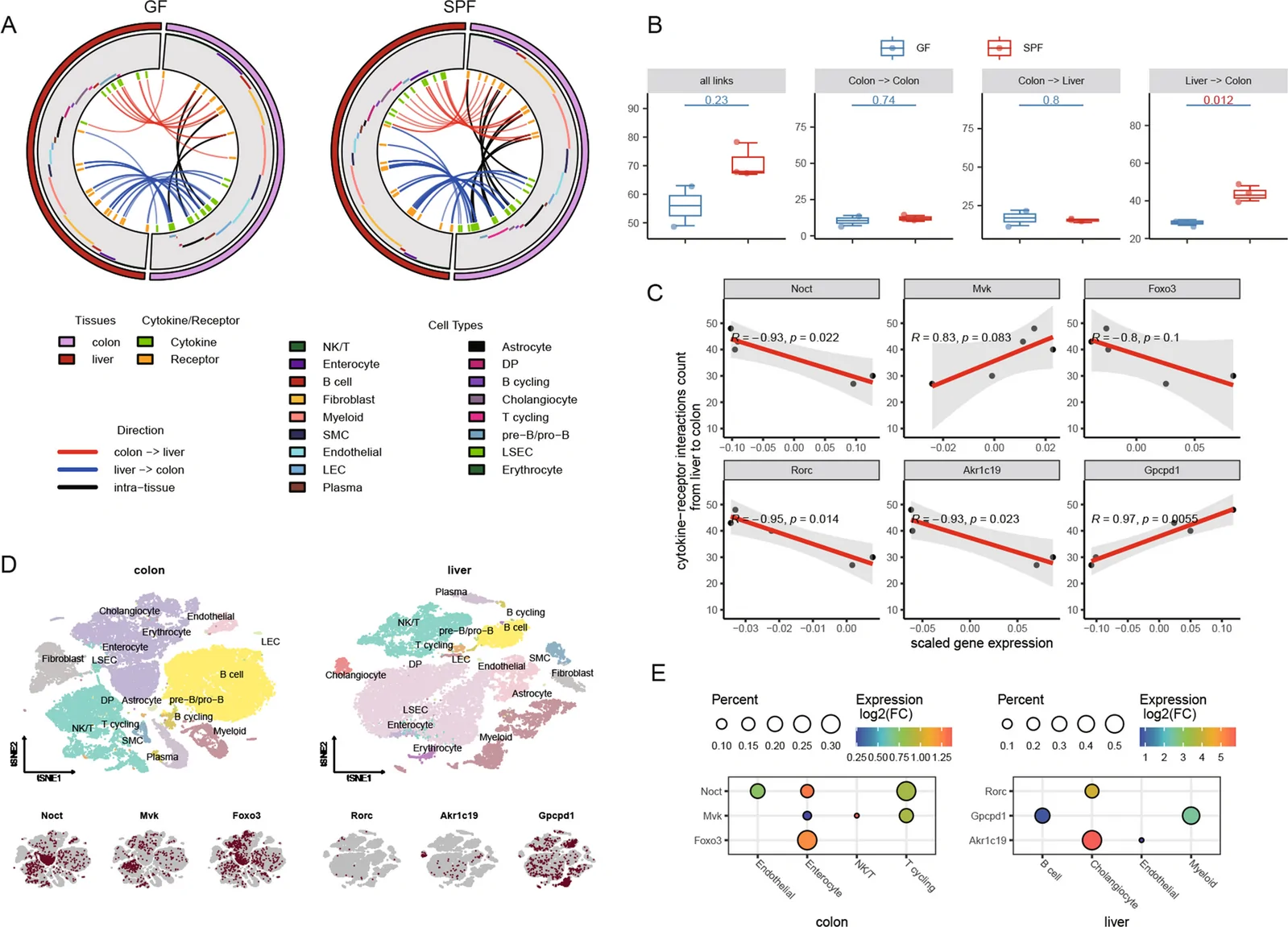
scRNA-seq reveals the effect of gut microbes on liver-gut axis. **A** Connection map of cytokine-receptor coupling across liver and colon in a representative GF (left) and SPF (right). Genes that code cytokines and receptors are aligned along the perimeter of the circles. From the outer layer to the center is the tissue expressing the genes inferred cell specificity, classification of cytokine or receptor, and the connections between cytokines and its matching receptors. The connections were indicated in different colors according to the classification of direction. **B** Links count of cytokine-receptor coupling of all direction. **C** Correlation between links count, which are from liver to colon, and expression level of top 3 genes in liver and colon, respectively. **D** t-distributed Stochastic Neighbor Embedding (tSNE) of scRNA-seq (top) and features plot of top3 genes (bottom). **E** Top 3 genes expression of each cell types in liver (right) and colon (left)

Emerging evidence indicates that the intestine, the body’s largest immune organ, engages in robust crosstalk with peripheral tissues, establishing various gut-peripheral organ axes. The gut-liver axis is considered pivotal among these. Leveraging public datasets (CNGBdb Project: CNP0005580; https://db.cngb.org/data_resources/project/CNP0005580/), we performed an initial exploration of the alterations in immune signal exchange along this axis under different environmental conditions (Fig. 5A), signal directionality along the axis was defined using known ligand-receptor pairs. Results show that enhanced liver-to-colon signaling was observed in SPF mice (Fig. 5B), whereas signals in other directions were unaltered, with no liver-to-liver transmission detected. Subsequently, we questioned if the key gut microbiota-responsive genes were involved in the signaling axis. This prompted a correlation analysis between the candidate microbiota-responsive genes expression and the signaling links (liver to colon), which revealed strong associations (|Cor|> 0.8). Among them, significant correlations were found for *Noct* (in colon, Cor = −0.93), *Rorc* (in liver, Cor = −0.95), and *Akr1c19* (in liver, Cor = −0.93), all showing strong negative correlations, while *Gpcpd1* (in liver, Cor = 0.97) exhibited a strong positive correlation (Fig. 5C). In addition, leveraging single-cell RNA sequencing data, we successfully mapped these key genes to specific cell types (Fig. 5D, E). Endothelial cells, enterocytes, and circulating T cells in the colon all expressed high levels of *Noct*. Besides, colonic enterocytes were found to express both 3 candidate microbiota‑responsive genes (*Noct*, *Mvk*, and *Foxo3*). In the liver, the expression of *Gpcpd1* primarily in B cells and myeloid cells, *Rorc* and *Akr1c19* were predominantly expressed in cholangiocytes. These suggest that colonic enterocytes and hepatic cholangiocytes are the primary responsive cell populations to gut microbiota in the colon and liver, respectively.

### scATAC-seq uncovers varied transcription initiation in colonic enterocytes

**Fig. 6:**
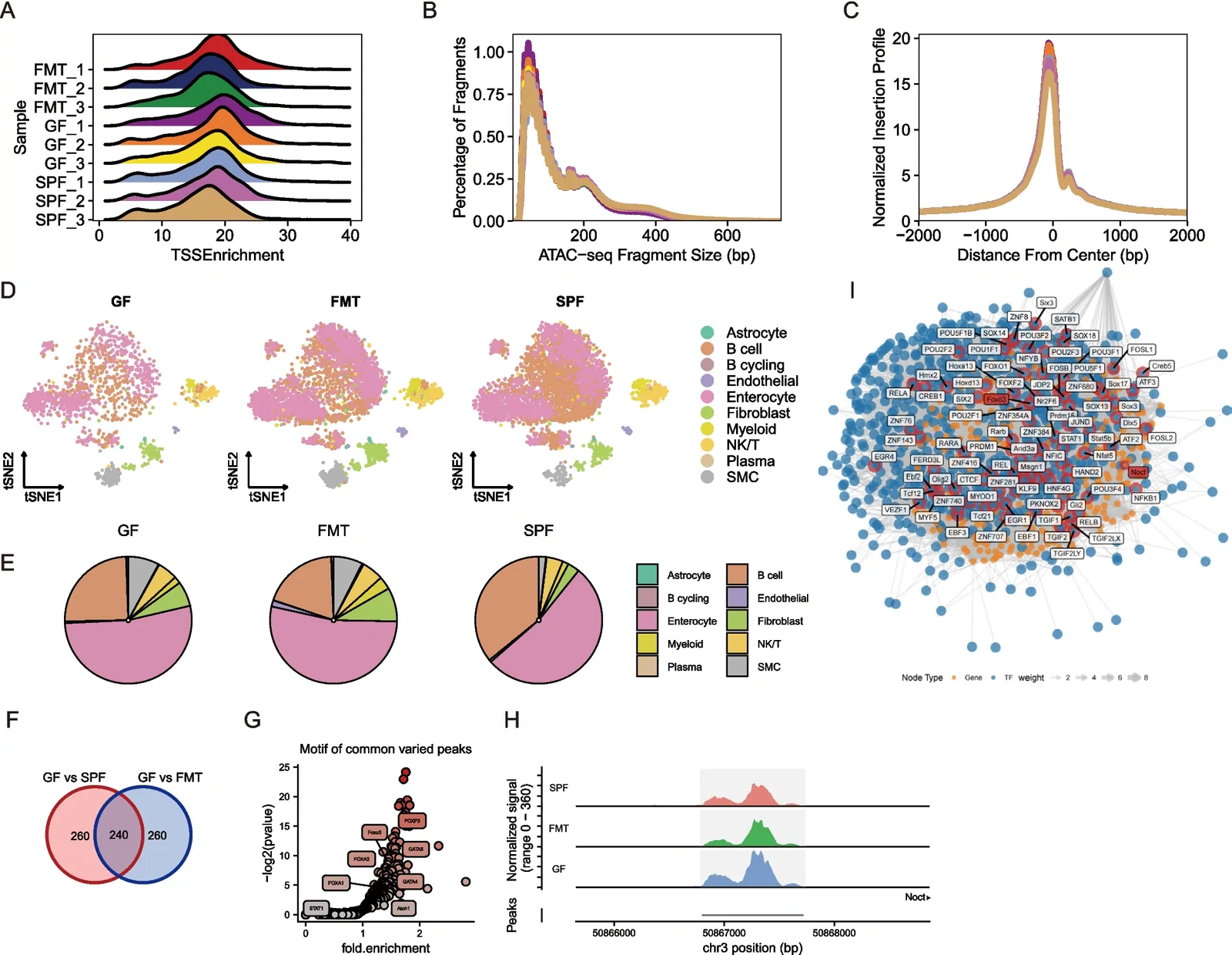
scATAC-seq uncovers varied transcription initiation in colonic enterocytes. **A–C** Quality of scATAC data, evaluated by TSS enrichment (**A**), fragment size (**B**) and distance from center (**C**). **D** t-distributed Stochastic Neighbor Embedding (tSNE) of scATAC-seq. **E** Ratio of all cell types in different conditions. **F** Intersection of significantly different peaks. **G** Motif significantly enriched in different peaks. **H** Different peaks plot of *Noct* in different conditions. **I** Network which consist of nearest gene of differential peaks position and motif of differential peaks. *Foxo3* and *Noct* are highlighted, and transcription factors of *Noct* are labeled

The aforementioned results indicate that the colon is the tissue most significantly influenced by the gut microbiota, especially colonic enterocyte. Previous studies indicate that gut microbiota can influence transcriptional initiation by modulating transcription factors via metabolites like butyrate. Given this, we analyzed single-nucleus ATAC-seq data to determine whether colonic gene expression differences originated from changes in transcription initiation. As a result, a total of 153.26 Gb size of data were produced, exhibiting strong signal specificity (Fig. 6A), proper nucleosomal patterning (Fig. 6B), and precise cleavage at open chromatin regions (Fig. 6C). Based on tSNE analysis, the total number of colon cells was lower in GF mice, but the proportional abundance of major cell types was largely consistent (Fig. 6D), except notable increased B cells upon colonization (Fig. 6E). Given that enterocytes constituted the dominant population (>50%) and were previously identified as the primary site of candidate microbiota-responsive genes expression, subsequent analyses were therefore focused on investigating chromatin accessibility differences specifically within the enterocytes compartment. Consequently, we identified 240 differentially accessible peaks that were significantly different in the enterocytes of both SPF and FMT mice (Fig. 6F). Motifs enriched in these differential peaks included binding sites for the transcription factor Foxo3 and other gut microbiota metabolite-responsive transcription factors, such as Foxp3, Stat1, and Atoh1 (Fig. 6G). Besides, down-regulated peaks, which included binding sites for 94 transcription factors, were detected in upstream of *Noct* in mice under NGF (Fig. 6H), corresponding to down-regulated expression level of *Noct* mRNA observed previously. At last, to gain a more intuitive understanding the regulatory relationships between genes chromatin accessibility and transcription factors, an integrated regulatory network was constructed by linking the transcription factors enriched at the differential peaks with their nearest genes (Fig. 6I). These reveal that gut microbiota reshape intestinal epithelial chromatin accessibility via specific transcription factors, thereby regulating host transcriptional programs and functional adaptation under symbiosis.

## Discussion

This study provides a comprehensive map of the gut microbiota’s influence on the host transcriptional landscape across six tissues. We found that the microbiota’s impact is highly tissue-specific, with the most profound effects observed in the colon and jejunum. While the presence of microbiota robustly enhanced immune-related pathways in the gut, its influence on peripheral tissues was more modulated, as evidenced by distinct gene expression patterns and remodeled ceRNA networks. By employing a machine learning strategy (LMM and LASSO regression), we precisely identified tissue-specific, microbiota-responsive genes. Our findings show that the gut microbiota exerts tissue‑specific effects on candidate microbiota‑responsive genes in non‑GF mice: it upregulates hepatic *Akr1c19* (human ortholog AKR1C1 associated with worse survival in LIHC), downregulates pulmonary *Cry1* (human ortholog associated with better survival in LUAD), and upregulates renal *Nampt* (human ortholog associated with worse survival in KIRC). These associations do not imply a functional contradiction but rather highlight the context‑dependent prognostic implications of microbiota‑regulated genes across different cancers. Furthermore, single-cell multi-omics analyses (scRNA-seq and scATAC-seq) elucidated the underlying mechanisms, pinpointing colonic enterocytes and hepatic cholangiocytes as primary responsive cell types. We further demonstrated that microbiota-driven changes in chromatin accessibility at transcription factor binding sites (e.g., for Foxo3) are linked to transcriptional alterations, particularly along the gut-liver axis. Collectively, our work unveils a complex, multi-layered regulatory network through which the gut microbiota fine-tunes host physiology in a tissue- and cell-type-specific manner, with implications for understanding disease pathogenesis.

A growing body of research in recent years has revealed that the gut microbiota may regulate peripheral tissues through its metabolites or membrane components—for instance, the presence of gut microbiota enhances adipocyte accumulation by promoting the expression of the FIAF protein [28–30], increases muscle mass [31], reduces blood–brain barrier permeability [32], and improves bone quality [33]. While recent multi‑omics studies (e.g., Shen et al., 2025) have examined the effects of microbiota depletion on organ morphology, immune homeostasis, and metabolism, a systematic transcriptome‑wide analysis including microbiota restoration (FMT) across multi tissues has not been reported. Our study therefore complements existing work by adding the dimensions of whole‑transcriptome profiling, ceRNA network remodeling, and FMT‑based restoration [23].

To distinguish direct from indirect targets of gut microbiota regulation among differentially expressed genes, we applied a machine learning-based screening approach. The linear mixed effects model (LMM) serves as a more reliable and flexible statistical inference tool in data screening. Particularly when dealing with small sample sizes, unbalanced data, or complex random effects, the application of Restricted Maximum Likelihood (REML) estimation combined with Kenward-Roger or Satterthwaite approximation methods significantly enhances the reliability of the results [34, 35], thereby establishing a solid foundation for rigorous screening of experimental data. In this study, we applied LMM with “tissue” and “housing environment” as mixed effects to screen for responsive genes. However, the LMM was unable to effectively distinguish between candidate microbiota-responsive genes resulting from the interaction of these two factors and those influenced by either factor alone, nor could it clarify the tissue specificity of gene expression. To address this, we subsequently performed Mfuzz clustering to group genes with consistent expression patterns across different tissues. This was followed by LASSO regression and random forest analysis to further refine the candidate genes, ultimately leading to the successful identification of representative candidate microbiota-responsive genes in each tissue. We acknowledge that the sample size for machine learning is modest; thus, the identified genes should be considered candidate effectors that require independent validation in larger cohorts.

The part of TCGA survival analysis, which associated candidate microbiota-responsive genes in mice with human cancer prognosis, do not imply direct causation from microbiota to human cancer outcomes, but they highlight potentially relevant genes for further investigation. Besides, none of the three colon‑specific candidate genes (*Foxo3*, *Txnrd1*, *Mvk*) showed significant survival associations in TCGA‑COAD. This may be due to several non‑mutually exclusive factors: (i) the intrinsic molecular heterogeneity of colon adenocarcinoma (e.g., consensus molecular subtypes CMS1‑4) could mask gene‑level survival effects; (ii) the relatively modest sample size of the COAD cohort (*n* = 461) for detecting small‑to‑moderate effect sizes; (iii) the possibility that microbiota‑responsive genes in the healthy murine colon do not directly translate to prognostic markers in established human colon cancer, where tumor‑intrinsic and microenvironmental changes may override microbiota‑driven expression patterns. Future studies with larger cohorts and subtype‑stratified analyses will be needed to clarify these observations.

The ceRNA regulatory network, operating through a “molecular sponge” mechanism [36, 37], enables precise gene expression control, functional coordination, and systemic robustness. It serves as a fundamental basis for cells to perform complex functions during essential physiological processes such as development, differentiation, homeostasis maintenance, and adaptation to environmental changes. Both the gut microbiota and the ceRNA regulatory network possess the ability to maintain their own homeostasis through self-regulation. It is intriguing to explore the potential regulatory interactions between these two systems. Our study offers initial insights into how the ceRNA network responds to the gut microbiota (further experimental validation, e.g., luciferase reporter assays, would be required to confirm true ceRNA functionality). Although several studies have touched upon this phenomenon [38, 39], a detailed mechanistic understanding of the microbiota’s contribution is still lacking, highlighting an important direction for future research.

Based on multi-tissue and multi-omics analyses, this study demonstrates that the gut microbiota orchestrates host transcriptional programs in a highly tissue-specific and cell-type-specific manner, extending beyond the gut to peripheral tissues. This multidimensional regulation involves differential gene expression, rewired ceRNA networks, and altered chromatin accessibility, collectively fine-tuning core physiological functions such as immunity and metabolism. Our findings provide a comprehensive atlas of microbiota-host interactions, offering novel insights into the mechanisms underlying microbiota-associated diseases.

## Conclusion

This study delineates the detailed mechanisms by which the gut microbiota orchestrates tissue-specific host transcription. Beyond reshaping immune and metabolic pathways, it remodels ceRNA networks, increasing non-coding RNA connectivity in gut tissues. Machine learning identified candidate microbiota‑responsive genes (e.g., hepatic *Akr1c19*, pulmonary *Cry1*) linked to cancer prognosis. Single-cell analyses pinpointed colonic enterocytes and hepatic cholangiocytes as primary responsive cells, demonstrating that microbiota-driven alterations in chromatin accessibility at transcription factor binding sites (e.g., for Foxo3) underlie transcriptional changes, particularly along the gut-liver axis. This integrated multi-omics atlas reveals how the microbiota fine-tunes host physiology through layered genomic regulation.

## Materials and method

### Experiment design

Eight week-old germ-free (GF) mice, germ-free mice undergoing fecal microbiota transplantation (FMT), and special-pathogen-free (SPF) mice were used, *n* = 3 each group. Fecal microbiota was obtained from age‑matched specific‑pathogen‑free (SPF) donor mice housed under the same facility. Time point after transplantation: FMT mice received fresh fecal suspension (200 µL, 0.1 g/mL in sterile PBS) by oral gavage daily for 3 consecutive days, and tissues were collected 4 weeks after the last gavage. Collecting almost 80 mg heart, liver, lung, kidney, colon and jejunum tissues for whole RNA sequencing. Sequencing were performed by Majorbio (Shanghai, China). Total 718 Gb raw data were obtained. Raw data in FASTQ format were firstly processed through in-house perl scripts to perform quality filtering. Filtered reads were obtained by removing low quality reads and reads containing adapters and ploy-Ns from the raw data. The filtered reads were mapped to mice reference genome (GRCm39.113) by using HISAT2 v2.2.1. Using featureCounts v2.0.1, statistical gene read counts were generated. Differently expressed genes (DEGs) were analyzed using R package DEseq2 v1.42.1, as *P* < 0.05, |fold change|> 1.2 were regarded as DEGs. Kyoto Encyclopedia of Genes and Genomes (KEGG) pathway enrichment were performed using R package clusterProfiler v4.10.1. All data visualizations were created using R version 4.3.1, for detailed code, see Code Availability.

### Linear mixed-effects model (LMM)

Gene expression data were normalized as reads per kilobase per million (RPKM), and genes with expression values greater than zero in at least five samples were retained for downstream analysis. To stabilize variance, expression values were transformed using log(1 + x). For each gene, we fitted a linear mixed‑effects model using the lmer function (R package lme4). The model formula was:

$$\begin{aligned} \mathrm{l}\mathrm{o}\mathrm{g}2(\mathrm{R}\mathrm{P}\mathrm{K}\mathrm{M}+1) \sim \mathrm{t}\mathrm{r}\mathrm{e}\mathrm{a}\mathrm{t}\mathrm{m}\mathrm{e}\mathrm{n}\mathrm{t} + \mathrm{t}\mathrm{i}\mathrm{s}\mathrm{s}\mathrm{u}\mathrm{e}\\ +\, \mathrm{t}\mathrm{r}\mathrm{e}\mathrm{a}\mathrm{t}\mathrm{m}\mathrm{e}\mathrm{n}\mathrm{t}:\mathrm{t}\mathrm{i}\mathrm{s}\mathrm{s}\mathrm{u}\mathrm{e} + (1 | \mathrm{m}\mathrm{o}\mathrm{u}\mathrm{s}\mathrm{e}\_\mathrm{i}\mathrm{d}) \end{aligned}$$

where treatment (GF, FMT, SPF) and tissue (colon, jejunum, liver, heart, lung, kidney) were included as fixed effects, along with their interaction. The term (1 | mouse_id) represents a random intercept for each individual mouse, accounting for the correlation among multiple tissues collected from the same animal. No random slopes were included, as likelihood ratio tests indicated that a random intercept alone sufficiently captured the within‑mouse dependence (*P* > 0.05). A two-way analysis of variance (ANOVA) was employed to test the interaction effect between treatment (GF, FMT, SPF) and tissue type on gene expression. For each gene, the following linear model was fitted:

$$\begin{aligned} Y &=\beta 0 +\beta 1 \cdot \mathrm{t}\mathrm{r}\mathrm{e}\mathrm{a}\mathrm{t}\mathrm{m}\mathrm{e}\mathrm{n}\mathrm{t}+\beta 2 \cdot \mathrm{t}\mathrm{i}\mathrm{s}\mathrm{s}\mathrm{u}\mathrm{e}+\beta 3\\ & \quad \cdot (\mathrm{t}\mathrm{r}\mathrm{e}\mathrm{a}\mathrm{t}\mathrm{m}\mathrm{e}\mathrm{n}\mathrm{t}\times \mathrm{t}\mathrm{i}\mathrm{s}\mathrm{s}\mathrm{u}\mathrm{e})+\epsilon \end{aligned}$$

where *Y* represents the gene expression level, *β*_0_ is the intercept, *β*_1_ and *β*_2_ denote the main effects of treatment and tissue, respectively, *β*_3_​ quantifies the interaction effect, and ϵ is the error term. Type III ANOVA was performed using the car package to assess the statistical significance of the interaction term. To control the false discovery rate (FDR), *P*-values were adjusted using the Benjamini–Hochberg method, with genes showing an FDR < 0.05 considered statistically significant. Model performance was evaluated using the coefficient of determination (R^2^), mean absolute error (MAE), and root mean square error (RMSE). Parallel computing via the future and furrr packages was implemented to enhance computational efficiency. All analyses were conducted in R (version 4.3.1) with the tidyverse, lme4, lmerTest, and car packages.

### Least Absolute Shrinkage and Selection Operator (LASSO)

Gene expression data were obtained from RNA-seq analysis of multiple tissues (colon, liver, jejunum, heart, lung, and kidney) from germ-free (GF, *n* = 3) and non-germ-free (NGF, *n* = 6) mice. Tissue-specific gene sets were predefined based on prior clustering analysis. Analyses were performed separately for each tissue using its respective tissue-specific gene set. We performed LASSO regression using the glmnet package in R with the following parameters: binomial family for binary classification, alpha = 1 for L1 regularization, and fivefold cross-validation to determine the optimal lambda value (lambda.min). Genes with zero variance were excluded, and remaining expression values were Z-score normalized prior to analysis. The response variable was coded as 0 for GF and 1 for NGF samples. Model performance was evaluated using accuracy and area under the receiver operating characteristic curve (AUC). Feature importance was determined by the absolute value of regression coefficients.

### Randomforest

Following LASSO feature selection, we performed Random Forest analysis using the randomForest package in R to evaluate the importance of identified key genes. The analysis was conducted separately for each tissue using their respective LASSO-selected gene sets. The Random Forest model was built with 500 trees (ntree = 500) and importance metrics were calculated based on Mean Decrease Accuracy. Gene expression data were used as predictors with treatment groups (GF vs NGF) as the response variable.

Gene name matching was implemented with a multi-step approach including exact matching, dash-to-dot conversion (to accommodate R’s column naming conventions), and partial matching to ensure maximum gene recovery from the expression matrix. Variable importance was visualized using bar plots showing Mean Decrease Accuracy for each gene, with genes ranked by importance value within each tissue. Results were compiled across all tissues to generate a comprehensive importance ranking of the 96 LASSO-selected genes.

### Survival analysis

Survival analysis using data from The Cancer Genome Atlas (TCGA) was performed to validate the clinical relevance of identified key genes. Four cancer types are involved, corresponding to tissue-specific gene sets: colon adenocarcinoma (TCGA-COAD), liver hepatocellular carcinoma (TCGA-LIHC), lung adenocarcinoma (TCGA-LUAD), and kidney renal clear cell carcinoma (TCGA-KIRC). Gene expression data (STAR-Counts) and clinical information were downloaded using the TCGAbiolinks package. For each cancer type, we analyzed the top 3 tissue-specific genes identified in machine learning analysis above. Survival analysis was conducted using the survival and survvminer packages. Patients were dichotomized into high and low expression groups based on median gene expression levels. Overall survival was assessed using Kaplan–Meier curves with log-rank tests for statistical comparison. Cox proportional hazards models were employed to evaluate the association between gene expression levels and survival outcomes.

### ceRNA network

To investigate the regulatory interactions between mRNAs, lncRNAs, and miRNAs across different tissues and treatment conditions, we constructed competing endogenous RNA (ceRNA) networks based on gene expression correlation. Note that these networks are based on expression correlations only; they do not include experimentally validated or predicted miRNA‑mRNA binding information. Therefore, they should be interpreted as correlational ceRNA interaction candidates rather than validated regulatory circuits. Gene expression data were filtered to include only tissue-specific genes identified from previous clustering analysis. For each tissue (colon, liver, jejunum, heart, lung, kidney) and treatment condition (GF, FMT, SPF), we performed Spearman correlation analysis using the psych package. Correlation coefficients and statistical significance were calculated with pairwise complete observations. Network edges were defined by stringent criteria: Spearman’s correlation coefficient > 0.4 and *P*-value < 0.05. Only interactions between different RNA biotypes (mRNA-lncRNA, mRNA-miRNA, lncRNA-miRNA) were retained to specifically capture potential ceRNA relationships. Self-loops and duplicate edges were removed to ensure network integrity. Node degree (connectivity) was calculated for each RNA molecule across conditions. Network visualization was performed using igraph, tidygraph, and ggraph packages. Degree distribution patterns were analyzed using density plots to compare network connectivity across different treatment conditions within each tissue.

### Single cell RNA-seq

scRNA-seq data were obtained from the public datasets CNGBdb (CNGBdb Project: CNP0005580; https://db.cngb.org/data_resources/project/CNP0005580/), which were derived from C57BL/6 J mice at 10 weeks of age, under GF and SPF housing conditions. Single-cell RNA sequencing data from liver and colon tissues were analyzed using Seurat (v4.0) to investigate the spatial expression patterns of tissue-specific candidate genes. Pre-processed Seurat objects containing quality-filtered cells with annotated cell types were subjected to scaling using the ScaleData function, followed by t-SNE dimensionality reduction based on the first 30 principal components. The expression of previously identified tissue-specific genes (*Noct*, *Mvk*, and *Foxo3* in colon; *Rorc*, *Akr1c19*, and *Gpcpd1* in liver) were analyzed using scaled expression values, calculating mean expression levels per biological sample across all cell types. Spatial expression patterns were visualized through t-SNE embeddings with graduated color scales, maintaining standardized visualization parameters for comparative analysis. A comprehensive, step-by-step protocol for the single-cell data analysis can be found in the official Seurat documentation (https://satijalab.org/seurat).

### Single cell ATAC-seq

scATAC-seq data were processed using the Signac package (v1.6.0) in R. Peak regions were called for each sample individually and merged to create a unified peak set. Dimensionality reduction was performed using latent semantic indexing (LSI), followed by UMAP visualization and clustering.

Cell type annotation was performed by transferring labels from a reference scRNA-seq dataset of colon tissue using canonical correlation analysis (CCA). We retained only high-confidence predictions (score > 0.7) for downstream analysis.

### Statistical methods

Statistical analyses were performed using R (version 4.3.1; R Development Core Team). A two-sided *P* value less than 0.05 was considered statistically significant. Continuous variables were expressed as mean ± standard deviation, while categorical variables were described as counts and percentages. Diagnostic performance of machine learning models was evaluated using the area under the receiver operating characteristic (ROC) curve, with additional metrics including sensitivity, specificity, accuracy, positive predictive value, and negative predictive value. Feature selection was performed using Least Absolute Shrinkage and Selection Operator (LASSO) regression with fivefold cross-validation, while variable importance was assessed through Random Forest analysis. Survival analysis employed Kaplan–Meier curves with log-rank tests and Cox proportional hazards models. Correlation analyses utilized Spearman’s rank correlation with a coefficient threshold of 0.4 and *P* < 0.05 for significance. Dimensionality reduction techniques including principal component analysis and t-distributed stochastic neighbor embedding (t-SNE) were implemented for data visualization. Network analyses were conducted using igraph and dependence packages, with node connectivity measured by degree centrality.

Based on multi-tissue and multi-omics analyses, this study demonstrates that the gut microbiota orchestrates host transcriptional programs in a highly tissue-specific and cell-type-specific manner, extending beyond the gut to peripheral tissues. This multidimensional regulation involves differential gene expression, rewired ceRNA networks, and altered chromatin accessibility, collectively fine-tuning core physiological functions such as immunity and metabolism. Our findings provide a comprehensive atlas of microbiota-host interactions, offering novel insights into the mechanisms underlying microbiota-associated diseases.

## Supplementary Information

Supplementary Material 1.

Supplementary Material 2.

Supplementary Material 3.

## Data Availability

The original contributions presented in the study are deposited in NCBI GEO (accession: GSE322803), publicly accessible without restrictions at https://www.ncbi.nlm.nih.gov/geo/query/acc.cgi?acc=GSE322803. No author contact or additional permission is needed, complying with the journal’s data policy. The complete code base and corresponding data for each figure can be accessed on the GitHub repository: [https://github.com/hillincre/Microbiota-disturbed-cross-tissue-transcriptome] (https:/github.com/hillincre/Microbiota-disturbed-cross-tissue-transcriptome).
