# Supplementary material for: Gut microbiota dysbiosis remodels the multi-tissue transcriptional landscape of the host
Source: Microbiome. 2026 Aug 14;14:248. doi: 10.1186/s40168-026-02495-6 (PMC13753458; doi:10.1186/s40168-026-02495-6)
Supplement: Supplementary file 1 [file 40168_2026_2495_MOESM1_ESM.docx]

**Supporting Information for：**

**Gut Microbiota Dysbiosis Remodels the Multi-Tissue Transcriptional Landscape of the Host**

Liang Huang^1,2,3^, Shuqi Liu^1,2,3^, Sai Xiao^1,2,3^, Yige Li^1,2,3^, Shiqi Luo^1,2,3^, Erjia Hou^1,2,3^, Yizhen Wang^1,2,3^ and Xin Zong^1,2,3,^ *

^1^ Key Laboratory of Molecular Animal Nutrition, Ministry of Education, College of Animal Sciences, Zhejiang University, 310058 Hangzhou, PR China.78

^2^ Key Laboratory of Animal Nutrition and Feed Science in Eastern China, Ministry of Agriculture, College of Animal Sciences, Zhejiang University, 310058 Hangzhou, PR China.

^3^ Zhejiang Key Laboratory of Nutrition and Breeding for High-quality Animal Products, College of Animal Sciences, Zhejiang University, 310058 Hangzhou, PR China.

***Correspondence**

Xin Zong, College of Animal Sciences, Zhejiang University, 310058 Hangzhou, PR China. Email: [zongxin@zju.edu.cn](mailto:zongxin@zju.edu.cn)

**Supplementary Figures (Fig. S1-S4)**

**Fig.S1** Significance test of principal component analysis (PCA).

**Fig.S2** Varied signal pathway caused by gut microbes. KEGG pathways enrichment of overlap genes, which showed consistent expression trend in tissues of mice under both FMT and SPF, compared to mice under GF.

**Fig.S3** LASSO regression path diagram and cross validation curve. Least Absolute Shrinkage and Selection Operator (LASSO) model parameter index, include path diagram, cross validation curve and area under curve (AUC).

**Fig.S4** ceRNA network of key effector genes. Alluvial maps show the correlations between mRNA, miRNA and lncRNA. Color of each RNA are fixed to enhance the visibility of differences.
